# Myositis ossificans of the masseter muscle: A rare location. Report of a case and review of literature

**DOI:** 10.4317/jced.52888

**Published:** 2016-04-01

**Authors:** Laia Fité-Trepat, Míriam Martos-Fernández, Margarita Alberola-Ferranti, Carolina Romanini-Montecino, Manel Saez-Barba, Coro Bescós-Atín

**Affiliations:** 1 MD. Resident, Oral and Maxillofacial Surgery Department, Vall d’Hebrón Hospital. Barcelona, Spain; 2 PhD, MD. Department of Pathology, Vall d’Hebrón Hospital. Barcelona, Spain; 3 MD. Resident, Pathology Department, Vall d’Hebrón Hospital. Barcelona, Spain; 4 MD, DDS. Assistant Surgeon, Oral and Maxillofacial Surgery Department, Vall d’Hebrón Hospital, Barcelona, Spain; 5 PhD MD, DDS. Head of Oral and Maxillofacial Surgery Department, Vall d’Hebrón Hospital. Barcelona, Spain. Researcher of the VHIR group

## Abstract

**Background:**

Myositis Ossificans is a rare heterotopic bone formation within a muscle being the masticatory muscles exceptionally involved. In most cases there is a previous trauma, bearing in mind that there may be many other etiologies. CT scan and panoramic radiographs along with histological findings are essential diagnostic aids.

**Case Desciption:**

We report a rare case of MO of masseter muscle in 49 years-old woman after repetitive wisdom tooth infection with the discussion of clinical, radiological and histological features.

**Clinical Implications:**

MO is a rare disease of masticatory muscles being the masseter the most frequently affected. Wide surgical excision with free margins is the treatment of choice although close postoperative monitoring it’s essential to avoid relapses.

** Key words:**Myositis ossificans, myositis ossificans traumatica, masticatory muscles, masseter muscle, trauma.

## Introduction

Myositis ossificans (MO) is also known as a Myositis ossificans traumatica (MOT) because in over half of the cases exists a direct relation with a single or repetitive injury of the muscle. The most accepted etiologic mechanism includes an osteoblast stimulation as a consequence of a bone or soft tissue damage causing a formation of new bone, dystrophic calcifications or calcified chondroid matrix. However, in approximately 25% of cases, there is no correlation with a trauma. Regardless of its origin, MO is a rare disease involving heterotopic ossification in the muscle or soft tissue (1-3).

The aim of this article is to report a case of a 49 year-old woman with MO located in the masseter muscle and a literature review about MO related to masticatory muscles due to the few reported cases.

## Case Report

**Figure 1:**
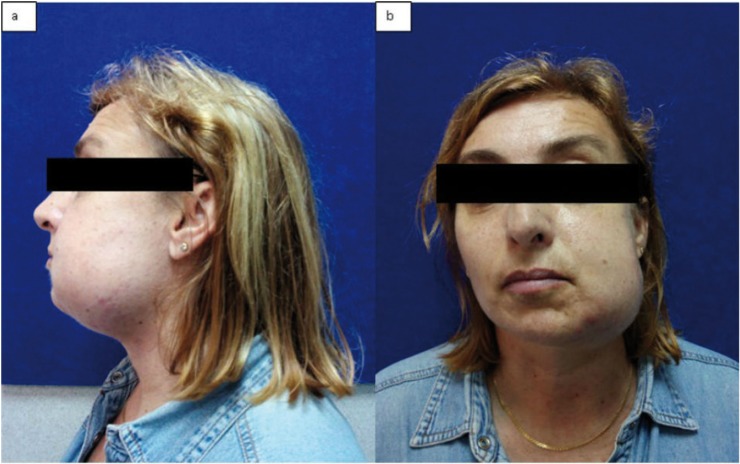
Preoperative images of 49 year-old female with a left paramandibular hard tumor accompanied by pain, swelling, progressive growth and difficulty in mouth opening of 10 days of evolution. a) left view b) frontal view.

**Figure 2:**
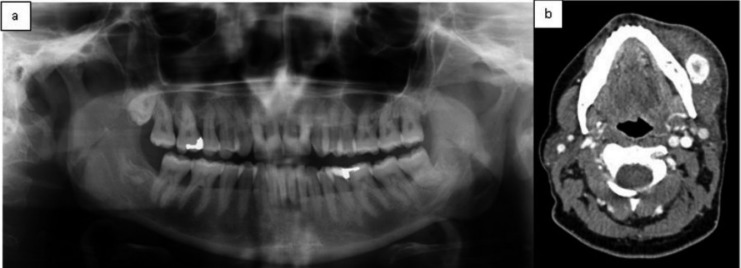
Radiological study. a) orthopantomography. b) CT scan: well-defined calcification located within masseter muscle.

**Figure 3:**
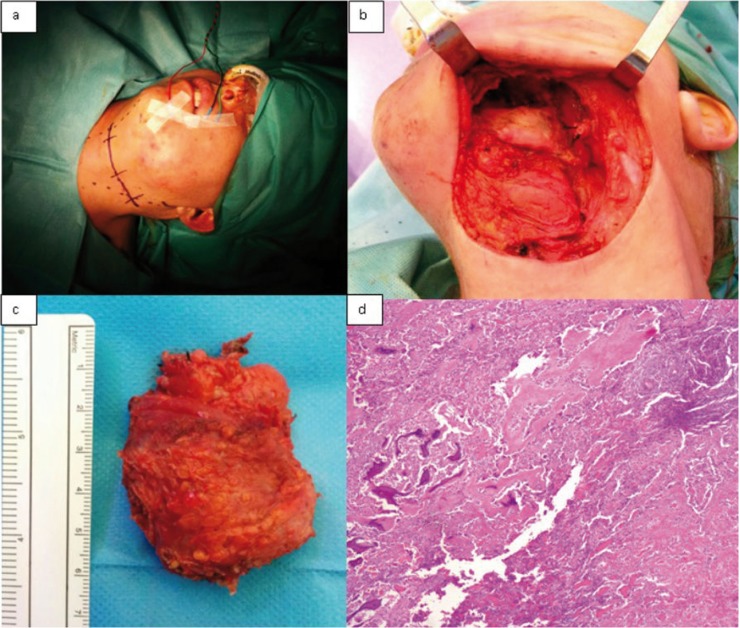
Intraoperative images. a) cervical approach b) identification of the tumor englobing the marginal nerve c) Macroscopic image of the surgical specimen (2,3 x 1,6 x 1,5cm) d) Histopathological examination showing a central zone of bone tissue surrounded by mature bone.

In April 2015, 49 years-old female was visited at Maxillofacial Surgery department of Vall d’Hebron Hospital to assess the sudden appearance of a left paramandibular hard tumor accompanied by pain, swelling, progressive growth and difficulty in mouth opening during last 10 days (Fig. 1). She denied any medical history of interest but referred repetitive infection of her left wisdom tooth few weeks ago. An orthopantomography and a CT scan were performed (Fig. 2) that showed a well-defined calcification within the left masseter muscle suggesting a benign tumor of soft tissue. Due to define its histology an incisional biopsy of the lesion was done. The anatomopathological study reflected morphologic changes compatibles with myositis ossificans. No malignant signs were observed in the sample. Surgical removal of the tumor under general anaesthesia was done by a left cervical approach (Fig. 3a). The lesion was exposed after a careful dissection and was excised, with 1cm of tumor-free margins, sacrificing the marginal nerve because it was totally surrounded by the MO (Fig. 3b). The definitive anatomopathological analysis described a central zone of bone tissue with abundant osteoblasts surrounded by mature bone tissue compatible with myositis ossificans (Fig. 3c-d). The edges of the sample were tumor free. At 3rd month postoperative the patient had no pain, a correct healing and a left marginal nerve paralysis.

## Discussion

MO is the most frequent extraskeletal bone forming in larger muscles of the legs (1), especially in the quadriceps femoris and brachialis anticus, however, its location in masticatory muscles is uncommon with only 52 reported since 1924 to current date (2,3). The longest series of cases has shown a male predominance (M 2,5:1 W) with a mean age of appearance between 38-48 years (2,3). Regarding the location, masseter muscle is the most affected, followed by medial and lateral pterygoid muscle, and finally, temporalis muscle in order of frequency (1,2). A suitable explanation for this incidence could be the external situation of the masseter that can easily receive direct extern trauma (2,4). Furthermore, lateral pterygoid and temporalis muscles tend to develop with synchronous lesions in other muscles (4). Despite trauma is considered to be the most likely etiology the exact mechanism of pathogenesis remains unclear. According to current data, a bone morphogenetic protein (BMP) signal from the point of injury induces a mesenchymal cells differentiation into osteoblasts or chondroblasts (3).

MO is classified into two groups: traumatic MO (MOT) and progressive MO (MOP) also called progressive fibrodysplasia ossificans (5). MOT is caused by traumatic etiologies, as dental extractions, repeated localized infections, local anaesthetic infiltrations - in particular in mandibular foramen and medial pterygoid muscle (4) -, direct or proximal physical trauma, temporomandibular joint luxation, neck immobilization; Literature reports that it might take over 3-6 weeks between the trauma and first manifestation of symptoms. MOP is an autosomal dominant genetic disease with variable expression and penetrance in which multiple heterotopic ossification develop systemically in various muscles, fascia, tendons and ligaments of the body. MOT has a good prognosis after a surgical treatment against MOP that produces a decreased survival due to respiratory muscles affection, and its predominance in teenagers or younger (1,6,7).

Trismus is the most frequent symptom of MO, as in our case (2,3). But other symptoms could be showed like pain, facial asymmetry or swelling. The absence of symptoms it’s also a possibility (8) and in some cases its diagnosis could be prolonged more than 20 years (9). A differential diagnosis should be done between others pathologies with similar clinical manifestations ( Table 1).

**Table 1:**
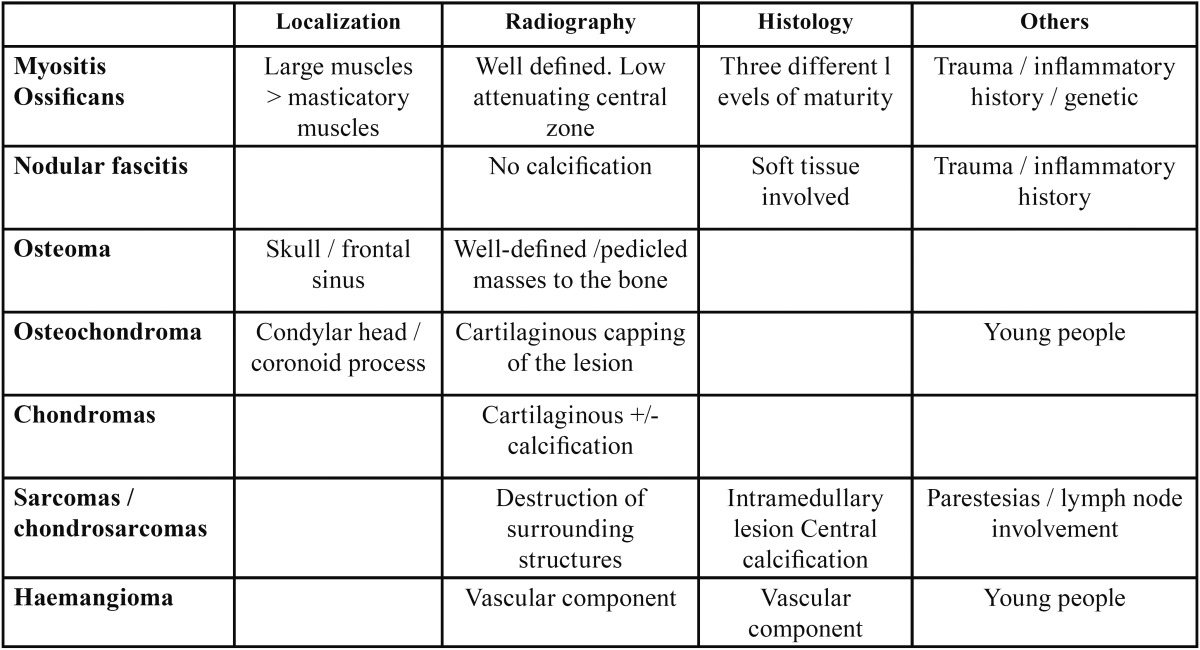
Clinical differential diagnosis.

Diagnosis is made by radiological and histological study. Although orthopantomography isn’t the most effective study to determinate the extension of the lesion, it may aid with the identification of odontogenic infection focus (3). CT scan is the most useful instrument which can show a well circumscribed image with high-attenuating periphery and a low-attenuating central portion within muscle or soft tissues. The histological examination highlights an inner zone of proliferating fibroblasts and mitotic activity, an intermediate zone of developing osteoid cells and collagen trabeculae and an outer zone of mature bone separated from the surrounding muscle by connective tissue without inflammatory infiltrate (1,4,5,10). The term myositis ossificans is contradictory because the inflammation is absent and, if present, it is usually minimal and the muscle may not be involved. It is important to make a differential diagnosis with extraosseous osteosarcoma and parosteal osteosarcoma where greater cytologic atypia and aberrant mitosis are present. The histological features of our patient included a lobulated, well delimited and partially encapsulated lesion with peripheral mature laminar bone that formed trabeculae. There was an intermediate area with osteoid material surrounded by osteoblasts and a central zone made of a cellular proliferation with mesenchymal features. Nor cytological atypia neither mitosis are seen in the lesion.

Surgical excision is the most consensual treatment, however, there are different opinions about if it is recommended an interval of 6 to 12 months after initial diagnosis before surgical treatment to allow a complete calcification of the lesion. On the other hand, about a third of cases resolve spontaneously, so it could be considered an expectant attitude (1). Some articles defend the use of interpositional materials, such as abdominal or buccal fat pads, with the aim of prevent relapse, hematoma or collapse after excision (1,6). Other authors have proposed a complementary treatment with drugs such as etidronate disodium -a biphosphonate commonly used as a prophylaxis and treatment of Paget’s disease which prevents aggregation, growth and calcification of crystals (4,7)-, nonesteroidal anti-inflammatory drugs, steroids, warfarin –a inhibitor of vitamin K products that reduces the production of osteocalcin which plays a significant role in metabolic regulation (1,6,11)-, and low-dose radiation. In our case, from diagnosis to definitive treatment we spent a total of 3 months and didn’t use any filling material or postoperative drug treatment. Nonetheless, a close monitoring of the patient is essential because the difficulty to establish a consensus about the most effective treatment due to the few published studies and their quality.
